# Release of monocyte migration signals by breast cancer cell lines after ablative and fractionated γ-irradiation

**DOI:** 10.1186/1748-717X-9-85

**Published:** 2014-03-26

**Authors:** Roman Hennel, Nikko Brix, Karin Seidl, Anne Ernst, Heike Scheithauer, Claus Belka, Kirsten Lauber

**Affiliations:** 1 Department of Radiation Oncology, Ludwig-Maximilians-University, Munich, Germany

**Keywords:** Ablative radiotherapy, Fractionated radiotherapy, Monocyte migration, Chemokinesis, Chemotaxis, Nucleotides, Dying cell clearance, Immunogenic cell death, Anti-tumor immunity

## Abstract

**Background:**

Radiotherapy, administered in fractionated as well as ablative settings, is an essential treatment component for breast cancer. Besides the direct tumor cell death inducing effects, there is growing evidence that immune mechanisms contribute - at least in part - to its therapeutic success. The present study was designed to characterize the type and the extent of cell death induced by fractionated and ablative radiotherapy as well as its impact on the release of monocyte migration stimulating factors by dying breast cancer cells.

**Methods:**

Cell death and senescence assays were employed to characterize the response of a panel of breast cancer cell lines with different receptor and p53 status towards γ-irradiation applied in a fractionated (daily doses of 2 Gy) or ablative setting (single dose of 20 Gy). Cell-free culture supernatants were examined for their monocyte migration stimulating potential in transwell migration and 2D chemotaxis/chemokinesis assays. Irradiation-induced transcriptional responses were analyzed by qRT-PCR, and CD39 surface expression was measured by flow cytometry.

**Results:**

Fast proliferating, hormone receptor negative breast cancer cell lines with defective p53 predominantly underwent primary necrosis in response to γ-irradiation when applied at a single, ablative dose of 20 Gy, whereas hormone receptor positive, p53 wildtype cells revealed a combination of apoptosis, primary, and secondary (post-apoptotic) necrosis. During necrosis the dying tumor cells released apyrase-sensitive nucleotides, which effectively stimulated monocyte migration and chemokinesis. In hormone receptor positive cells with functional p53 this was hampered by irradiation-induced surface expression of the ectonucleotidase CD39.

**Conclusions:**

Our study shows that ablative radiotherapy potently induces necrosis in fast proliferating, hormone receptor negative breast cancer cell lines with mutant p53, which in turn release monocyte migration and chemokinesis stimulating nucleotides. Future studies have to elucidate, whether these mechanisms might be utilized in order to stimulate intra-tumoral monocyte recruitment and subsequent priming of adaptive anti-tumor immune responses, and which breast cancer subtypes might be best suited for such approaches.

## Background

Radiotherapy is a crucial treatment part for the management of breast cancer. Commonly, it is applied in daily fractions of 1.8-2 Gy for 5 to 7 weeks to a total dose of 50 to 66 Gy [1-5]. Fractionated irradiation schemes are considered to be beneficial for the reduction of tumor burden, since they are intended to exploit the discrepant DNA repair capacity of tumor and normal tissue. Larger, irreparable damage per unit dose is induced in the tumor, whereas efficient DNA repair mechanisms compensate for the damage induced by the daily irradiation fractions in the adjacent normal tissue. Moreover, tumor reoxygenation can occur between the fractions and tumor cells can redistribute to more radiosensitive phases of the cell cycle [6]. At present several clinical trials support the use of higher doses per fraction in order to shorten the overall treatment period. In the UK doses of 2.66 Gy per fraction are already accepted practice [7,8], and ongoing trials will determine in how far even higher single doses (5.7-6 Gy) may define a new optimum. Additionally, there are specific applications, where ablative, large single doses of 10–25 Gy are delivered locally to the tumor, for instance during intra-operative radiotherapy (IORT) [9-11].

The tumor cell death inducing effect of radiotherapy has been regarded as the major determinant of its therapeutic success for a long time. Nevertheless, there is accumulating experimental evidence that innate as well as adaptive immune responses contribute - at least in part - to the reduction of tumor burden and tumor control [12]. Essentially, previous studies have revealed that radiotherapy stimulates a type I interferon-dependent priming of adaptive anti-tumor immune responses, including tumor-specific CD8^+^ cytotoxic T cells, by antigen-presenting cells (APCs) [13-17]. These effects were only observed in case of ablative but not fractionated radiotherapy, and the underlying mechanisms remain largely elusive. Conceivably, tumor cells respond differently to γ-irradiation when applied in an ablative or a fractionated setting. The mode as well as the extent of cell death might vary substantially. And since different types of cell death are well known to have different immunological consequences, we proposed that the extent as well as the type of tumor cell death in response to radiotherapy might govern and shape the subsequent anti-tumor immune responses [18]. For systemic anthracycline therapy it has been reported that danger signals released from dying tumor cells trigger the activation of APCs and subsequent T cell priming [19]. One of the initial steps in this scenario was the intra-tumoral recruitment of monocytic precursor cells, which then differentiated into highly potent APCs [20]. Therefore, the present study was designed to analyze the cell death response of different breast cancer cell lines towards fractionated and ablative radiotherapy and its impact on the release of signaling molecules, which stimulate monocyte migration. We observed that fast proliferating, hormone receptor negative, and p53 mutant cell lines predominantly underwent necrosis in response to radiotherapy, particularly when administered in an ablative regime. Necrosis induction was paralleled by the release of apyrase-sensitive nucleotides, which effectively stimulated monocyte migration and chemokinesis. In hormone receptor positive breast cancer cells with functional p53 this process was impaired by irradiation-induced surface expression of the ectonucleotidase CD39 that degrades extracellular nucleotides. Hence, our study opens the question, whether ablative radiotherapy might be utilized for targeted necrosis induction in fast proliferating, hormone receptor negative breast cancer with defective p53 in order to stimulate intra-tumoral monocyte recruitment and subsequent priming of adaptive anti-tumor immune responses.

## Methods

### Cells and reagents

The human breast cancer cell lines MCF7, BT474, HCC1937, HCC1806, MDA-MB468, and BT549 were obtained from ATCC (Manassas, VA, USA) or CLS (Heidelberg, Germany), and were cultured in RPMI-1640 medium supplemented with 10% heat-inactivated fetal calf serum (FCS), 100 units/ml penicillin, 0.1 mg/ml streptomycin, and 10 mM HEPES (all from Life Technologies, Karlsruhe, Germany) at 37°C and 5% CO_2_ (MCF7, BT474, HCC1937, and HCC1806), or in DMEM (BT549), or DMEM/F12 (1:1) medium (MDA-MB468) supplemented with 10% heat-inactivated FCS, 100 units/ml penicillin, and 0.1 mg/ml streptomycin at 37°C and 7.5% CO_2_, respectively.

THP-1 cells were obtained from ATCC and were cultured in RPMI-1640 medium supplemented with 10% heat-inactivated FCS, 100 units/ml penicillin, 0.1 mg/ml streptomycin, and 10 mM HEPES. Preparation of human peripheral blood monocytes was carried out as described previously [21]. Briefly, PBMCs were prepared from heparinized blood of healthy volunteers by Biocoll density gradient centrifugation (Biochrom AG, Berlin, Germany). Monocytes were positively selected from PBMCs with anti-CD14 magnetic beads (Miltenyi, Bergisch Gladbach, Germany) according to the manufacturer’s recommendations and allowed to recover for 1 day in X-Vivo 15 medium (Lonza, Basel, Switzerland) supplemented with 10% autologous serum, 100 units/ml penicillin, and 0.1 mg/ml streptomycin before further use.

The p53 status of all breast cancer cell lines was determined by cDNA sequencing. Full length PCR products were generated from cDNA (80 ng per reaction) by employing 5 units HotStar HiFidelity DNA-Polymerase in 1 × HotStar HiFidelity reaction buffer, and 1 × Q solution (all from Qiagen, Hilden, Germany) in the presence of 1 μM of each primer (p53 Forward 5′-ATG GAG GAG CCG CAG TCA G-3′, p53 Reverse 5′-TCA GTC TGA GTC AGG CCC TTC T-3′, synthesized by Sigma-Aldrich, Taufkirchen Germany) in 100 μl final volume (cycling program: 1 × 5′ 95°C; 40 × 15″ 95°C, 1′ 60°C, 1′30″ 72°C; 1 × 10′ 72°C). The amplicons were purified by the NucleoSpin Extract II Kit (Macherey & Nagel, Dueren, Germany), and sequencing was performed by Seqlab Sequencing Services (Goettingen, Germany).

Carbobenzoxy-valyl-alanyl-aspartyl-[O-methyl]-fluoromethylketone (zVAD-fmk) was obtained from Bachem (Bubendorf, Switzerland), necrostatin-1 from Enzo Life Sciences (Loerrach, Germany), calcein-AM from Merck Calbiochem (Darmstadt, Germany), bafilomycin A1 and ARL-67156 from Tocris R&D Systems (Wiesbaden, Germany). The annexin V-FITC apoptosis detection kit, anti-CD39-PE, anti-CD73-FITC, and anti-CD203c-APC antibodies were purchased from BD Biosciences (Heidelberg, Germany), and 5-dodecanoylaminofluorescein-di-β-galactopyranoside (C12-FDG-FITC) was from Life Technologies.

Mouse monoclonal westernblot antibodies anti-p21^WAF1^ and anti-vinculin were obtained from BD Biosciences or Sigma-Aldrich, respectively. The chemokines SDF-1α and WKYMVm (agonist of formyl-peptide receptors 1, 2, and 3) were from R&D Systems, adenosine 5′-triphosphate disodium salt (ATP) from Sigma-Aldrich, and nucleotide diphosphohydrolase (apyrase) was purchased from New England Biolabs (Frankfurt, Germany).

### Growth analyses and determination of doubling times

Cells were seeded into 24-well plates (2.5 × 10^4^ cells per well) and allowed to adhere for 5 h. Medium was replaced (medium supplemented with 10% or 2.5% FCS was used as indicated), and cells were grown for up to 4 days. Every day, cells were harvested by trypsinization and cell numbers were determined by counting. Growth curves were generated by plotting log cell number (y-axis) versus time (x-axis), and doubling times were calculated on the basis of the slopes of the corresponding regression lines.

### X-ray treatment and production of cell-free culture supernatants

Cells were seeded into 6-well (0.5-1 × 10^6^ cells per well) or 24-well plates (0.25-1 × 10^5^ cells per well) in culture medium supplemented with 10% FCS and allowed to adhere overnight. Immediately prior to irradiation, culture medium was replaced by serum-reduced medium (2.5% FCS). Cells were irradiated at the indicated doses with a Mueller RT-250 γ-ray tube (200 kV and 10 mA, Thoraeus filter, 1 Gy in 1 min 52 s). Fractionated irradiation was carried out daily. Cell-free supernatants were collected by centrifugation (10,000 g, 5 min, 4°C) at the indicated time points and stored at -80°C until further use.

### SDS-PAGE and Westernblot analyses

Reducing 6-15% gradient SDS-PAGE and Westernblot analyses of whole cell lysates were performed as described previously [22,23] with 300 μg protein extract per lane. After electrophoretic separation, proteins were transferred to PVDF Immobilon FL membranes (Merck Millipore, Darmstadt, Germany). Membranes were blocked with 5% low-fat milk in TBST buffer (13 mM Tris–HCl pH 7.5, 150 mM NaCl, and 0.02% Triton X-100) and incubated with monoclonal mouse antibodies against p21^WAF1^ (BD Biosciences) or vinculin (Sigma-Aldrich), respectively. After incubation with the corresponding IRDye-conjugated secondary antibodies (LI-COR Biosciences, Bad Homburg, Germany) and extensive washing in TBST buffer, IRDye fluorescence was read with a LI-COR Odyssey scanner.

### Flow cytometric measurement of phosphatidylserine externalization, plasma membrane integrity, senescence-associated β-galactosidase activity, and ectonucleotidase surface expression

All FACS measurements were performed on an LSRII cytometer (BD Biosciences) and data were analyzed with FACSDiva (BD Biosciences) or FlowJo 7.6.3 Software (Tree Star Inc., Ashland, OR, USA), respectively.

Externalization of phosphatidylserine (PS) and plasma membrane integrity were measured by staining with annexin V-FITC/propidium iodide (annexin V staining kit, BD Biosciences) as described previously [24]. Briefly, 1 × 10^5^ cells were incubated with 5 μl FITC-labeled annexin V in 50 μl staining buffer (both from BD Biosciences) supplemented with 5 μg/ml propidium iodide (PI, Sigma-Aldrich) for 30 min on ice. Following an additional washing step in staining buffer, annexin V-FITC and PI fluorescence were assessed by flow cytometry. Cells with positive annexin V-FITC but negative PI signal were considered apoptotic, and cells double-positive for annexin V-FITC and PI staining were considered necrotic. In order to distinguish primary from secondary necrotic cells, the poly-caspase inhibitor carbobenzoxy-valyl-alanyl-aspartyl-[O-methyl]-fluoromethylketone (zVAD-fmk, Bachem) was employed. zVAD-fmk blocks apoptosis and the transition into secondary necrosis. Hence, annexin V-FITC/PI double positive cells, which were detected in the presence of zVAD-fmk, were considered primary necrotic.

Senescent cells were stained by using 5-dodecanoylaminofluorescein-di-β-galactopyranoside (C12-FDG-FITC, Life Technologies), a fluorogenic substrate of senescence-associated β-galactosidase [25]. At the indicated time points after irradiation, cells were incubated with 100 nM bafilomycin A1 (Tocris R&D Systems) in serum-free medium for 1 h at 37°C for lysosomal alkalinization. Subsequently, C12-FDG-FITC was added at a final concentration of 50 μM, and cells were incubated for 1 h at 37°C to allow substrate conversion. After two washing steps in PBS, cells were collected by trypsinization and analyzed by flow cytometry. Cells with high C12-FDG-FITC and high SSC signal were considered senescent.

For ectonucleotidase surface staining, 1 × 10^5^ cells were incubated with 2 μl anti-CD39-PE, anti-CD73-FITC, or anti-CD203c-APC in 50 μl FACS staining buffer (all from BD Biosciences) for 30 min on ice. After two washing steps in FACS staining buffer, cells were analyzed by flow cytometry. Relative surface expression was calculated as the median fluorescence intensities of anti-ectonucleotidase staining subtracted by the corresponding isotype controls.

### Transwell migration assay

Transmigration assays were performed in 96-well Multiscreen-MIC transwell chambers with 5 μm pore size (Merck Millipore) as described before [22,23]. In brief, 1 × 10^5^ calcein-labeled THP-1 cells per well were seeded in a final volume of 80 μl onto the 96-well filter Plate. 320 μl of supernatants or chemokines dissolved in serum-free RPMI-1640 medium were added to the lower chamber. The filter was mounted onto the lower chamber and transmigration was allowed for 90 min at 37°C. Subsequently, the cells in the lower chamber were collected by centrifugation and lysed in 100 μl lysis buffer (20 mM HEPES-K pH 7.4, 84 mM KCl, 10 mM MgCl_2_, 0.2 mM EDTA, 0.2 mM EGTA, 0.5% Igepal). Green calcein fluorescence was quantified with a Synergy MX fluorescence reader (BioTek Instrumtents GmbH, Bad Friedrichshall, Germany), and transmigration was calculated as percentage of total cells deployed.

In some experiments, supernatants were subjected to ultrafiltration with VivaSpin 2 centrifuge tubes with an exclusion limit of 10 kDa (Sartorius Stedim Biotech, Goettingen, Germany) as described before [22]. After passing the entire liquid phase through the filter, the filter was rinsed well with culture medium and the volume of the two fractions (substances smaller and larger than 10 kDa) was readjusted to the initial volume employed. Then, the fractions were applied to a transmigration assay.

Apyrase treatment was carried out by adding 500 milliunits of nucleotide diphosphohydrolase (apyrase, New England Biolabs) to 1.5 ml culture supernatant and allowing nucleotide degradation for 30–50 min at 37°C. Heat-inactivated apyrase served as a control. After digestion, supernatants were used in transwell migration or chemotaxis/chemokinesis assays, respectively.

### Chemotaxis/chemokinesis assay in IBIDI μ-slide 2D chemotaxis chambers

IBIDI μ-slide 2D chemotaxis chambers (IBIDI, Munich, Germany) were used to analyze chemotaxis and/or chemokinesis of primary human monocytes by live cell tracking as described before [22]. Briefly, monocytes were seeded into the observation area of the chamber in X-Vivo 15 medium supplemented with 5% autologous serum. Adherence was allowed for 15 min, and non-adherent cells were carefully washed away. The reservoirs of the chamber were filled with medium supplemented with 5% autologous serum, and the stimulus was added to the upper reservoir. The slide was mounted onto the heated stage of an AxioObserver Z1 inverted microscope (Zeiss, Goettingen, Germany), and time-lapse video microscopy was performed at 37°C and 5% CO_2_ for 3 h at 5 × magnification. Pictures were taken every 2 min and migration of 40 randomly picked cells was tracked with the ImageJ manual tracking plug-in. Accumulated distance, euclidean distance (linear distance between start and end position), and y forward migration index (yFMI = mean of (endpoint in y direction/accumulated distance) of all cells analyzed) were determined with the IBIDI chemotaxis and migration tool (IBIDI). Analysis window was set from 10 min to 2 h and 10 min (2 h time frame). Apyrase treatment of culture supernatants was performed as described for transwell migration assays.

### Quantitative Realtime RT-PCR

RNA isolation and qRT-PCR analyses were performed as described previously [26,27]. Briefly, total RNA was extracted with the NucleoSpin RNA II Kit (Macherey & Nagel). 1 μg of isolated RNA was subjected to reverse transcription with 200 units RevertAid reverse transcriptase in the presence of 50 μM random hexamers, 5 μM Oligo(dT)_18_, 400 μM dNTPs, and 1.6 units/μl Ribolock RNase inhibitor (all from Fermentas, St. Leon-Rot, Germany). The resulting cDNA (20 ng per reaction) was applied to qRT-PCR analyses (20 μl final volume) with 300 nM primers (synthesized by Sigma-Aldrich) in 1× Maxima SYBR Green qPCR Mastermix (Fermentas) and a standard cycling protocol (10 min 95°C, 45× (15 s 95°C, 30 s 60°C)) on an LC480 qPCR cycler (Roche Applied Science, Penzberg, Germany). The following primer pairs were used: p21^WAF1^ Forward 5′-CTG GAG ACT CTC AGG GTC GAA A-3′, p21^WAF1^ Reverse 5′-AGT GGT AGA AAT CTG TCA TGC TGG T-3′, Egr-1 Forward 5′-GAG CAC CTG ACC GCA GAG TC-3′, Egr-1 Reverse 5′-CCA GCA CCT TCT CGT TGT TCA-3′, 18S rRNA Forward 5′-CGG CTA CCA CAT CCA AGG AA-3′, 18S rRNA Reverse 5′-GCT GGA ATT ACC GCG GCT-3′, β2-microglobulin Forward 5′-TGC TCG CGC TAC TCT CTC TTT C-3′, β2-microglobulin Reverse 5′-TCT CTG CTG GAT GAC GTG AGT AAA C-3′. Relative quantification was performed by employing the standard curve method, and the results were normalized on the means of 18S rRNA and β2-microglobulin. Untreated control cells were used as calibrator.

## Results and discussion

**Figure 1:**
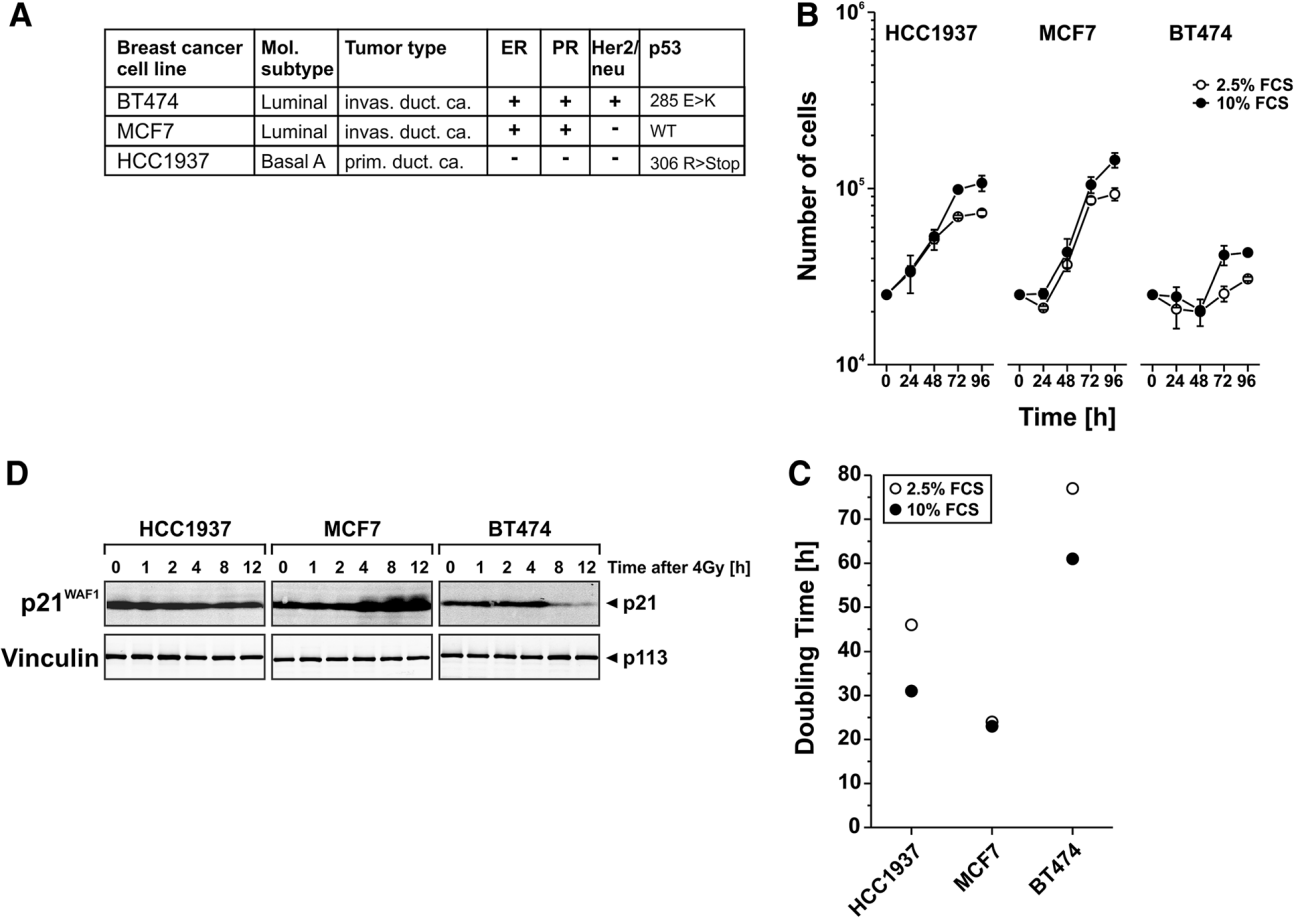
**Breast cancer cell lines of different origin reveal different doubling times and p53 functionality. (A)** Breast cancer cell lines used in the present study. Molecular subtype, tumor type and receptor status have previously been reported [29], and p53 mutational status was determined by cDNA sequencing. **(B)** Growth curves of breast cancer cell lines were generated in the presence of 2.5% or 10% FCS, respectively. Means ± s.d. of triplicates are shown. **(C)** Doubling times of exponentially growing cells were calculated from the data shown in **(B)**. **(D)** p21^WAF1^ induction as an indicator of p53 functionality was examined in whole cell lysates after irradiation with 4 Gy at the indicated time points by 6-15% SDS-PAGE (300 μg protein extract per lane) and subsequent immunoblot analysis. Vinculin served as a loading control.

A few previous studies have shown that radiotherapy can stimulate anti-tumor immune reactions, which contribute to the reduction of tumor burden [13-17]. In principle, the authors observed a type I interferon-dependent, APC-mediated priming of tumor-specific CD8^+^ T cell responses. Notably, the induction of these T cell responses was limited to ablative radiotherapy regimes, where γ-irradiation was applied at high single doses of more than 10 Gy. The tumor cell response towards low or high single doses as well as fractionated γ-irradiation in terms of apoptosis, necrosis, and senescence induction is likely to diverge substantially, and different types of tumor cell death are well known to stimulate different types of immunological consequences. Therefore, we hypothesized that the type of tumor cell response, which is induced by different regimes of radiotherapy, might shape the immunological consequences [18,28]. In order to address this issue, we analyzed the tumor cell response towards different regimes of γ-irradiation in three breast cancer lines: HCC1937, MCF7, and BT474. We deliberately chose cell lines of divergent molecular breast cancer subtypes as well as divergent estrogen, progesterone, and Her2/neu receptor status (Figure 1A) [29,30]. The cell lines exhibited clear differences in proliferation rates with short (MCF7, 24 h), intermediate (HCC1937, 46 h), and long (BT474, 77 h) doubling times in the presence of 2.5% FCS (Figure 1B, C). p53 mutation status and function were confirmed by cDNA sequencing and by immunoblot analysis of p21^WAF1^ induction in response to γ-irradiation at 4 Gy. Only MCF7 cells, which have been reported to possess wildtype p53 [30], revealed irradiation-induced upregulation of p21^WAF1^ protein expression starting approximately 4 h and reaching a plateau around 8 h after irradiation.

### Different γ-irradiation regimes induce different modalities of cell death and senescence in breast cancer cell lines

**Figure 2:**
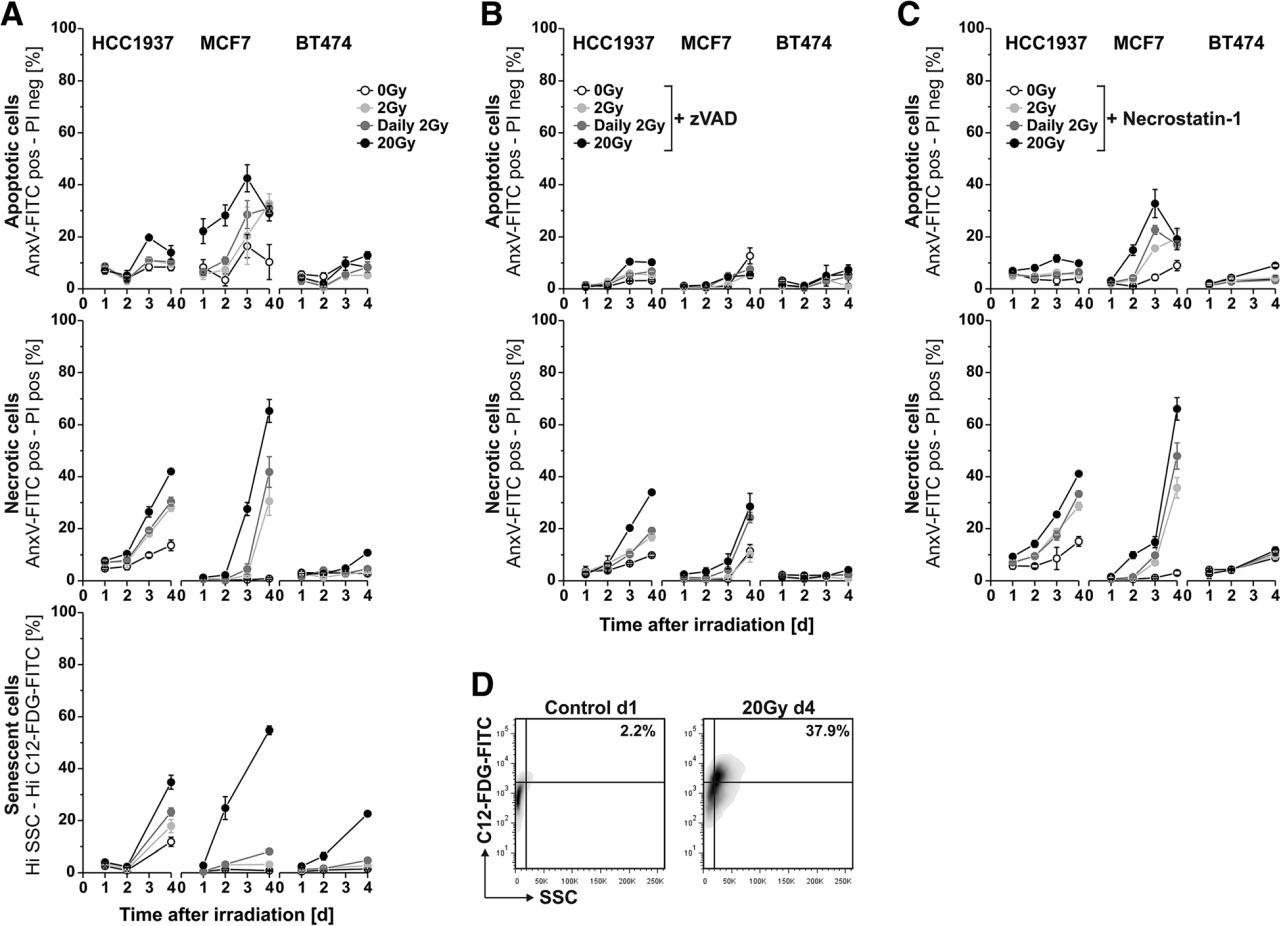
**Different γ-irradiation regimes induce different modalities of cell death and senescence in breast cancer cell lines. (A)** Induction of apoptosis, necrosis, and senescence. Breast cancer cell lines were left untreated or γ-irradiated at single doses of 2 Gy, 20 Gy, or daily fractions of 2 Gy, respectively. Induction of apoptosis and necrosis was determined 1–4 days after irradiation by annexin V-FITC/PI staining and FACS analysis. Annexin V-FITC positive, PI negative cells were considered apoptotic, double positive cells were considered necrotic. Senescence induction was measured by flow cytometric SA-β-gal staining with the fluorogenic substrate C12-FDG-FITC. Cells with high C12-FDG-FITC and high SSC signal were considered senescent. Means ± s.d. of triplicates are depicted. **(B)** Induction of apoptosis and necrosis in the presence of zVAD-fmk. PS externalization and plasma membrane integrity were measured as in **(A)** in the presence of 50 μM of the poly-caspase inhibitor zVAD-fmk. Means ± s.d. of triplicates are shown. **(C)** Induction of apoptosis and necrosis in the presence of necrostatin-1. PS externalization and plasma membrane integrity were measured as in **(A)** in the presence of 50 μM of the necroptosis inhibitor necrostatin-1. Means ± s.d. of triplicates are shown. **(D)** Representative dot plots of HCC1937 cells stained for SA-β-gal activity with the fluorogenic substrate C12-FDG-FITC.

Next, we investigated the type of tumor cell response towards different regimes of γ-irradiation. Cells were irradiated at single doses of 2 Gy or 20 Gy, or daily fractions of 2 Gy, respectively, and the percentage of apoptotic, necrotic, and senescent cells was measured by flow cytometry over a period of 4 days after irradiation (Figure 2A, D). In order to distinguish between primary and secondary (post-apoptotic) necrosis, we employed the poly-caspase inhibitor zVAD-fmk, which blocks apoptosis and the subsequent transit into secondary necrosis (Figure 2B). The necroptosis inhibitor necrostatin-1 was used to assess the contribution of necroptosis in our experimental system (Figure 2C). We made the following observations: (i) The strongest response of apoptosis, necrosis, and senescence induction was detected in fast proliferating MCF7 and HCC1937 cells. Slowly proliferating BT474 cells revealed only a moderate degree of apoptosis, necrosis, or senescence induction, respectively. (ii) Fast proliferating, p53 wildtype MCF7 cells underwent a combination of apoptosis and necrosis – primary as well as secondary (post-apoptotic) necrosis. Appearance of senescent MCF7 cells was only observed in response to ablative irradiation with 20 Gy. (iii) HCC1937 cells with mutant p53 predominantly underwent primary, apoptosis-independent necrosis and senescence. (iv) Of all γ-irradiation regimes applied, ablative irradiation at 20 Gy provoked the most pronounced cellular responses in terms of apoptosis, necrosis, and senescence induction. These findings allow the conclusion that ionizing irradiation - particularly when applied in an ablative setting of high single doses - primarily induces cell death and senescence in fast proliferating cancer cells. In cells with wildtype p53, a combination of apoptosis, primary, and secondary (post-apoptotic) necrosis is the major consequence, whereas cells lacking functional p53 mainly undergo primary necrosis and senescence. The contribution of necroptosis in this context is only a marginal one. These findings are in line with reports showing that p53 is crucial for irradiation-induced apoptosis, either via transcriptional activation of pro-apoptotic p53 target genes, including Bax, Puma, and Noxa, or via transcription-independent pathways, respectively [31-35]. In addition, p53 has been reported to be essential for establishing and maintaining certain types of senescence [36,37]. Nevertheless, p53-independent forms of senescence apparently do exist [38].

### Ablative γ-irradiation induces the release of low molecular weight, apyrase-sensitive factors, which stimulate monocyte chemokinesis

**Figure 3:**
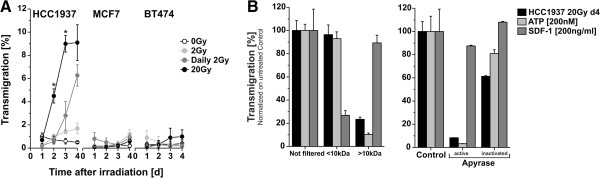
**Ablative γ-irradiation induces the release of low molecular weight, apyrase-sensitive factors, which stimulate THP-1 cell migration. (A)** THP-1 cell transwell migration. Breast cancer cells were left untreated or γ-irradiated as in Figure 2. Cell-free supernatants were collected 1–4 days after irradiation and applied to transwell migration assays with THP-1 cells. Means ± s.d. of quadruplicates are given. Asterisks indicate p < 0.05 as determined by unpaired Student’s *t*-test analysis (20 Gy vs. daily 2 Gy). **(B)** The transmigration stimulating factors are of low molecular weight and sensitive to apyrase treatment. Supernatants of HCC1937 cells irradiated at 20 Gy were collected on day 4 after irradiation and subjected to ultrafiltration with Vivaspin 2 columns (Molecular weight cut-off 10 kDa) or apyrase treatment (33.3 milliunits active or heat-inactivated apyrase/ml, 30 min at 37°C). Culture medium supplemented with ATP (200 nM) or SDF-1α (200 ng/ml) was treated in parallel. Afterwards, transwell migration assays with THP-1 cells were performed and the percentage of transmigrated cells was normalized on the corresponding untreated control. Means ± s.d. of quadruplicates are shown.

In order to stimulate productive, tumor-specific immune responses by radiotherapy, irradiated, dying tumor cells have to be detected and engulfed by APCs, which subsequently migrate into the draining lymph nodes, process and (cross-) present ingested tumor-antigens, and thus finally prime adaptive anti-tumor immune responses, including tumor-specific CD8^+^ cytotoxic T cells [18,39,40]. The initial step in this scenario is the recruitment of APCs by dying tumor cells - either tissue resident APCs or circulating monocytic precursors, which in turn can give rise to dendritic cells or macrophages, respectively. In order to examine the process of monocyte migration in the context of different radiotherapeutic regimes, we collected cell-free supernatants of HCC1937, MCF7, and BT474 cells 1–4 days after γ-irradiation with single doses of 2 Gy or 20 Gy, or daily fractions of 2 Gy, and applied them to transwell migration assays with monocytic THP-1 cells (Figure 3A). A clear and time-dependent migratory response was detected with supernatants of HCC1937 cells, which had been ablatively irradiated at a single dose of 20 Gy. Significantly reduced, yet still well detectable was THP-1 cell migration towards supernatants of HCC1937 cells, which had been subjected to the fractionated irradiation scheme with daily doses of 2 Gy. Of note, the migration stimulating capacity of HCC1937 supernatants paralleled necrosis induction and preceded the onset of senescence in HCC1937 cells (Figure 2) suggesting that necrotic and not senescent cells were the source of monocyte migration signals. Along this line, it was not surprising that supernatants of irradiated BT474 cells, which revealed very little necrosis induction even in response to irradiation with 20 Gy (Figure 2), did not significantly stimulate monocyte migration (Figure 3A). However, MCF7 cells despite extensively undergoing primary and secondary necrosis in response to ablative irradiation with 20 Gy as well as fractionated irradiation with daily doses of 2 Gy (Figure 2), did also not release detectable amounts of monocyte attraction signals (Figure 3A). Before addressing this issue in greater detail, we focused on the process of monocyte attraction by irradiated, necrotically dying HCC1937 cells.

Different necrotic cell-derived danger signals have been reported to be involved in monocyte recruitment. High molecular weight compounds, like heat shock proteins, high-mobility group box 1 protein (HMGB-1), S100 protein family members, small nuclear ribonucleoproteins, monosodium urate crystals, or nucleic acids, as well as low molecular weight compounds, like nucleotides, have been described [41]. In order to elucidate, which of these factors might contribute to monocyte attraction by ablatively irradiated HCC1937 cells, cell-free supernatants were subjected to ultrafiltration with an exclusion limit of 10 kDa. THP-1 cell migration towards the filtered supernatants was virtually not affected after high molecular weight compounds had been removed. Comparable results were obtained for purified ATP (MW = 507 Da), whereas the classical CXC chemokine SDF-1α (MW = 11 kDa) was more or less completely retained in the fraction with a molecular weight of more than 10 kDa, thus confirming a proof-of-principal of this procedure (Figure 3B left panel). Furthermore, incubation with active but not heat-inactivated nucleotide diphosphohydrolase (apyrase) completely abrogated THP-1 cell migration towards supernatants of HCC1937 cells that had been irradiated at 20 Gy. Again, parallel results were observed for purified ATP, whereas migration towards SDF-1α was basically not impaired by apyrase digestion (Figure 3B right panel). These findings allow the conclusion that the THP-1 cell migration stimulating factors, which are released by ablatively irradiated, necrotically dying HCC1937, are of low molecular weight and sensitive to apyrase treatment, apparently nucleotides. Different studies have previously provided evidence for the involvement of extracellular nucleotides in the recruitment of monocytes, macrophages, and dendritic cells by dying cells *in vitro* and *in vivo*[20,42,43]. However, currently it is being controversially discussed, whether nucleotides per se do stimulate directional chemotactic responses in monocytes and macrophages, or if they rather act as auto- and paracrine amplifiers of other chemotactic stimuli, such as complement C5a [44,45].

**Figure 4:**
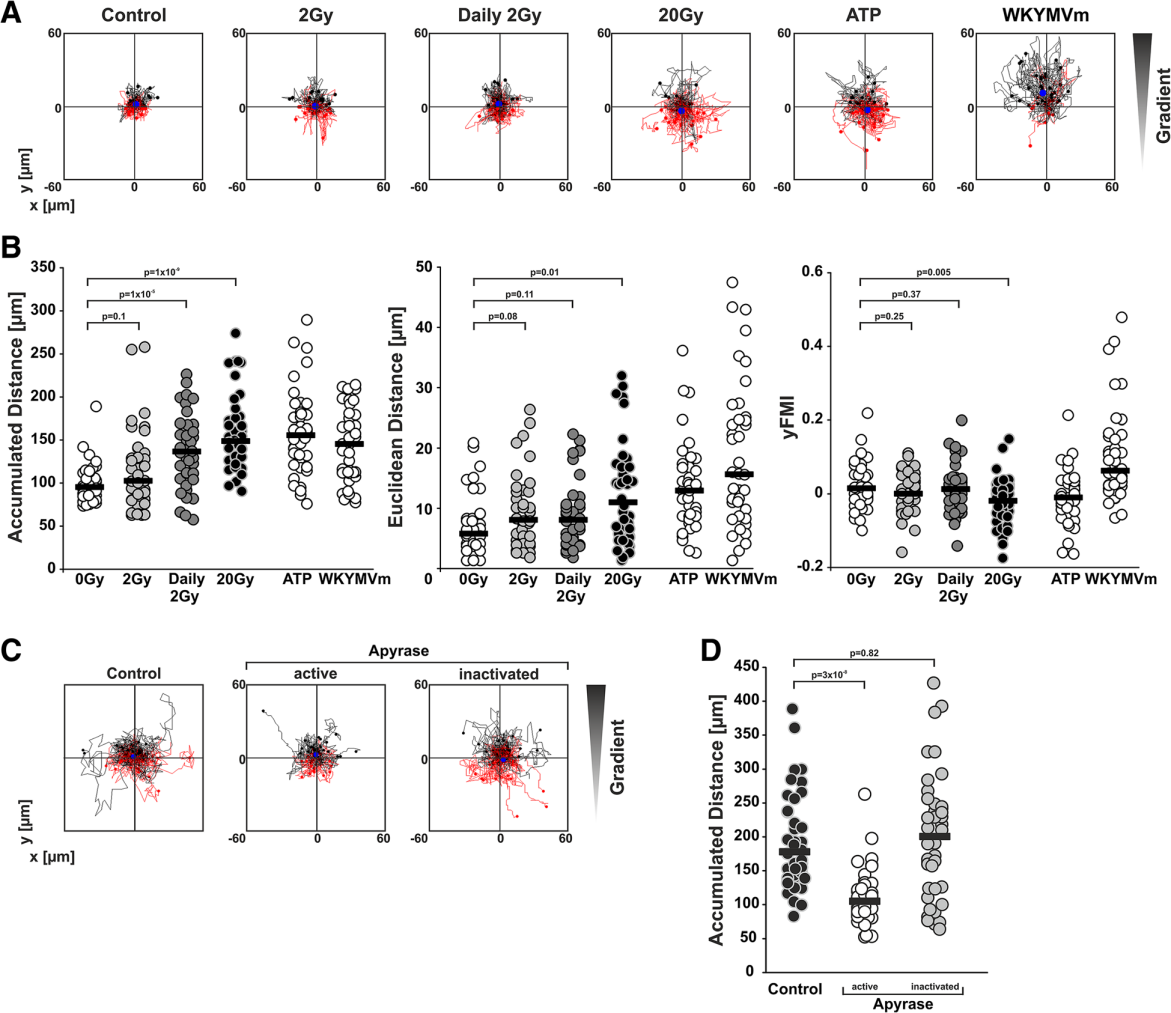
**Apyrase-sensitive nucleotides derived from dying cells stimulate monocyte chemokinesis. (A)** Chemotaxis/chemokinesis of primary human monocytes. HCC1937 cells were treated as in Figure 3A, supernatants were harvested on day 4 after irradiation, and chemotaxis/chemokinesis of primary human monocytes was analyzed by live cell tracking in IBIDI μ-slide chemotaxis 2D chambers. ATP (1 μM) and the FPR agonist WKYMVm (1 μg/ml) served as controls. Trajectory paths of 40 randomly picked cells are shown. Black paths depict cells with net migration upwards, red paths depict cells with net migration downwards. The filled blue circle represents the center of mass after 2 h of migration. **(B)** Parameters of chemotaxis/chemokinesis. The trajectory paths of 40 randomly picked cells as shown in **(A)** were analyzed for accumulated distance, euclidean distance (linear distance between start and end position), and the forward migration index in y-direction of the gradient (yFMI = mean of (endpoint in y direction/accumulated distance) of all cells analyzed). Analysis window was set from 10 min to 2 h 10 min (2 h time frame). Bars indicate the median values of 40 cells analyzed, and p-values were calculated by unpaired Student’s *t*-test. **(C)** The chemokinesis stimulating factors are sensitive to apyrase treatment. Supernatants of HCC1937 cells irradiated at 20 Gy were collected on day 4 after irradiation and incubated with active or heat-inactivated apyrase (33.3 milliunits apyrase/ml, 50 min at 37°C). Then, they were applied to chemotaxis/chemokinesis assays with primary human monocytes as in **(A)**. Trajectory paths of 40 randomly picked cells are shown. **(D)** Accumulated distance of the plots shown in **(C)**. Bars depict the median values of 40 cells analyzed, and p-values were calculated by unpaired Student’s *t*-test.

Therefore, we next characterized the migratory response of primary human monocytes towards supernatants of γ-irradiated HCC1937 cells by time-lapse video microscopy in 2D chemotaxis/chemokinesis chambers. The obtained trajectory paths and the detailed analysis of accumulated and euclidean distance as well as the forward migration index in direction of the gradient (yFMI) clearly show that the migratory response of primary monocytes towards supernatants of ablatively irradiated HCC1937 cells was a chemokinetic and not a chemotactic one (Figure 4A, B). In comparison to the untreated controls, supernatants of HCC1937 cells irradiated at 20 Gy intensified and accelerated monocyte migration as revealed by significant increases in the accumulated as well as the euclidean distance. However, monocyte migration was not directed towards the chamber, in which the supernatant was applied, and the yFMI value was even negative. Interestingly, parallel results were obtained for purified ATP supporting the conclusion that in our system nucleotides do not stimulate chemotaxis but rather chemokinesis as has already been described by others [44]. For comparison, the chemotactic FPR agonist WKYMVm was employed. Here, the increase in the accumulated distance was comparable to the one obtained with supernatants of ablatively irradiated HCC1937 cells and ATP, but the acquired euclidean distance was noticeably higher, and the yFMI was clearly positive, as the vast majority of cells migrated in the direction of the gradient. Notably, supernatants of HCC1937 cells that had been subjected to the fractionated irradiation scheme with daily doses of 2 Gy stimulated monocyte chemokinesis to a lesser, yet in terms of the accumulated distance still significant extent. Chemokinesis in the presence of supernatants collected from HCC1937 irradiated at a single dose of 2 Gy did not differ from the untreated control. Again, apyrase treatment significantly reduced monocyte chemokinesis stimulated by supernatants of ablatively irradiated HCC1937 cells, and the median accumulated distance declined to the level that was observed with supernatants of viable control cells (Figure 4B, C, D).

Hence, our migration data clearly show that necrotically dying, ablatively irradiated HCC1937 cells - and to a lesser extent also cells subjected to fractionated irradiation with daily doses of 2 Gy - release low molecular weight, apyrase-sensitive nucleotides, which stimulate monocyte chemokinesis in a similar fashion as ATP.

### Ablative γ-irradiation induces the upregulation of CD39 surface expression in MCF7 breast cancer cells

**Figure 5:**
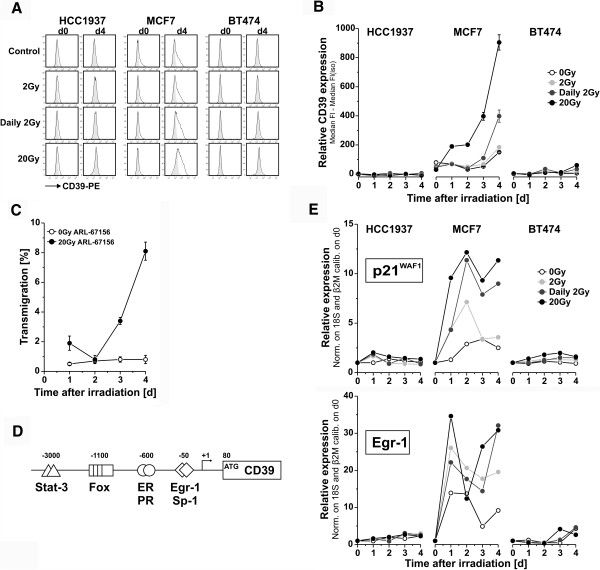
**A blative** γ**-irradiation induces the upregulation of CD39 surface expression in MCF7 breast cancer cells. (A)** CD39 surface expression on d0 and d4. Breast cancer cells were irradiated as indicated, collected by trypsinization, and CD39 surface expression was analyzed on d0 and d4 after irradiation by flow cytometry. Representative histograms are shown (black lines represent CD39 staining, filled grey areas the corresponding isotype controls). **(B)** Time course of CD39 upregulation. Cells were irradiated as indicated and CD39 surface expression was analyzed on d0-d4 after irradiation. Relative CD39 surface expression was calculated as the median fluorescence intensities of anti-CD39 staining subtracted by the corresponding isotype controls. Means ± s.d. of triplicates are shown. **(C)** Pharmacological inhibition of CD39 ectonucleotidase results in the release of monocyte migration stimulating factors by ablatively irradiated MCF7 cells. MCF7 cells were irradiated at 20 Gy or left untreated as in Figure 3A. Then, the CD39 inhibitor ARL-67156 was added at a final concentration of 100 μM and refreshed daily. The collected culture supernatants were applied to a transwell migration assay with THP-1 cells. Means ± s.d. of quadruplicates are given. **(D)***In silico* analysis of the human CD39 promoter. Binding sites for nuclear hormone receptors (ER, PR), Egr-1, and others, including Sp-1, Stat-3 and members of the forkhead transcription factor family (Fox), were identified. **(E)** Analysis of p21^WAF1^ and Egr-1 mRNA expression in response to different irradiation regimes. Cells were irradiated as in **(B)**, and 0–4 days after irradiation p21^WAF1^ and Egr-1 mRNA levels were determined by qRT-PCR analysis. Results were normalized on the means of 18S rRNA and β2-microglobulin, and untreated cells (d0) served as calibrator. Means of duplicates are given.

In contrast to HCC1937 cells, supernatants of MCF7 cells did not stimulate monocyte migration, although MCF7 cells strongly underwent primary and secondary necrosis in response to ablative γ-irradiation with 20 Gy (Figure 2). Complex nucleotide secretion processes, like the caspase/pannexin axis, which is activated during apoptosis and has been described to be impaired in MCF7 cells [46], apparently are of minor importance in case of necrosis-associated nucleotide release, since during necrosis the plasma membrane disintegrates and intracellular contents can passively leak out. Hence, the question that arises is: why do supernatants of necrotic MCF7 cells not contain monocyte migration stimulating nucleotides? A conceivable explanation for this observation would be that MCF7 cells express ectonucleotidases, which degrade extracellular nucleotides [47]. Surface staining of ectonucleotidases revealed that this in fact was the case (Figure 5A, B and data not shown). In contrast to HCC1937 and BT474 cells, MCF7 cells showed a low, but well detectable basal expression of the ectonucleotidase CD39, which was strongly increased in response to irradiation with 20 Gy and to a lesser extent also by fractionated irradiation with daily doses of 2 Gy (Figure 5A, B). Importantly, pharmacological inhibition of CD39 ectonucleotidase activity by addition of ARL-67156 resulted in the release of comparable amounts of THP-1 cell migration stimulating factors by ablatively irradiated MCF7 cells as had been observed with HCC1937 cells (Figure 5C and Figure 3A). Thus, upregulated CD39 apparently degrades extracellular nucleotides released by necrotically dying MCF7 cells.

The irradiation-induced increase in CD39 surface expression revealed a biphasic kinetics with an initial rise between days 1 and 2 after irradiation and an even stronger increase starting on day 3. The basal expression of CD39 in MCF7 cells has already been reported by others, but the mechanisms, which account for the differences in CD39 expression compared to HCC1937 and BT474 cells, are poorly understood [48]. Candidate transcriptional regulators in this regard are p53 and the nuclear hormone receptors for estrogen (ER) and progesterone (PR), since the three breast cancer lines differ in p53 functionality and hormone receptor status (Figure 1A). *In silico* analysis of the CD39 promoter region employing the AliBaba 2.1 platform (http://www.gene-regulation.com/pub/programs/alibaba2/index.html) revealed several transcription factor binding sites, including sites for the estrogen receptor (ER) and the progesterone receptor (PR) but no p53 response element (Figure 5D). Yet, p53- and ER-mediated transcriptional regulation appear to be closely interconnected, since they do not only mutually regulate each other’s expression but also have been described to control target gene expression in a coordinate manner [49-52]. Hence, p53 and ER might orchestrate basal CD39 expression in MCF7 cells. Following γ-irradiation, particularly when applied in an ablative scheme, MCF7 cells showed a robust activation of p53 as revealed by induction of p21^WAF1^ mRNA and protein expression (Figure 5E, Figure 1D). Hence, activated p53 (in cooperation with ER) might account for the upregulation of CD39 expression, since it was only observed in MCF7 cells and the induction of the prototypical p53 target p21^WAF1^ displayed a comparable biphasic time course as that of CD39. Nevertheless, indirect mechanisms, including the p53-mediated activation of other transcriptional regulators, could also be involved. As such Egr-1, an immediate early response transcription factor, which is well known to be induced and activated by ionizing irradiation and whose response element was identified close to the transcription start site within the CD39 promoter (Figure 5D), was induced in γ-irradiated MCF7 cells in a similar fashion as p21^WAF1^ and CD39 [53] (Figure 5E). Interestingly, Egr-1 has been reported to interact with p53 and to enhance transcriptional activation by p53 [54,55]. Our data do not allow detailed conclusions on the mechanisms, which govern irradiation-induced upregulation of CD39 expression in MCF7 cells. Nevertheless, they support a scenario, in which p53, ER and Egr-1 could play a crucial role. Further studies are required to elucidate this issue in greater depth and to find out if other transcription factors, such as Sp-1, Stat-3 or NF-kB, also are involved. It should be noted that we also measured the surface expression levels of CD73 and CD203c, two other well-known ectonucleotidases, but we did not detect any basal expression, nor an irradiation-induced upregulation in the three breast cancer cell lines tested (data not shown).

### Fast proliferating breast cancer cells with mutant p53 and a strong necrosis response towards ablative γ-irradiation release factors that stimulate monocyte migration

**Figure 6:**
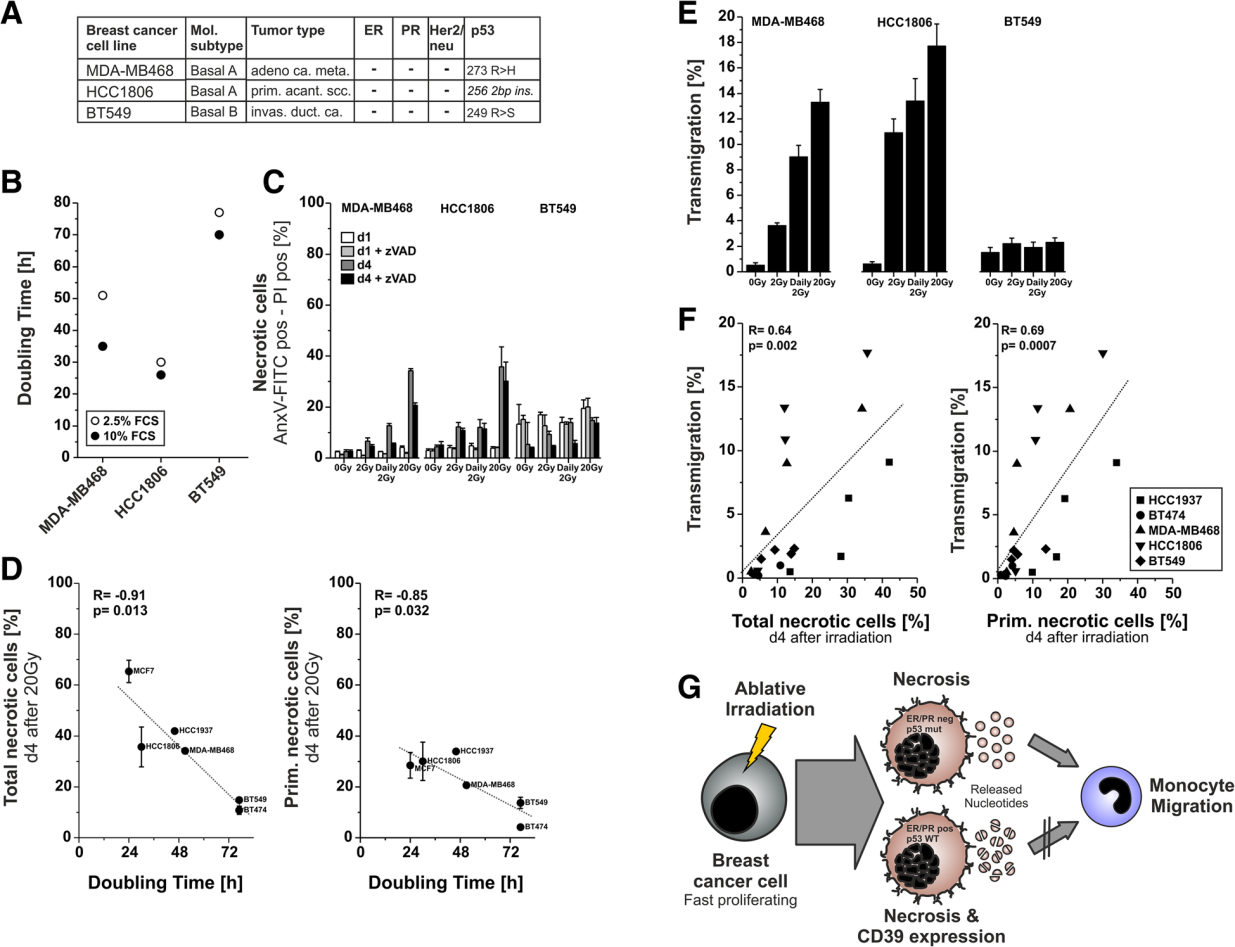
**Fast proliferating breast cancer cells with mutant p53 and a strong necrosis response towards ablative γ-irradiation release factors that stimulate monocyte migration. (A)** Breast cancer cell lines used. Tumor subtype and receptor status have previously been reported [29]. p53 mutational status was determined by cDNA sequencing. HCC1806 gave no p53 PCR product, but the mutation in codon 256 was reported before [30]. **(B)** Growth curve analysis in the presence of 2.5% or 10% FCS. **(C)** Induction of necrosis in response to irradiation. Cells were irradiated as indicated ± 50 μM zVAD-fmk. PS externalization and plasma membrane integrity were determined as in Figure 2. Double positive cells were considered necrotic. Means ± s.d. of triplicates are shown. **(D)** Fast proliferating cells reveal a stronger necrosis response towards ablative γ-irradiation. Pearson correlation analysis of doubling times (Figures 1C and 6B) and necrosis (Figures 2A,B and 6C) was performed. The percentage of total necrotic cells or primary necrotic cells was employed. **(E)** THP-1 cell migration. Cell-free supernatants were collected 4 days after irradiation and applied to transwell assays as in Figure 3. Means ± s.d. of quadruplicates are given. **(F)** p53 mutant cell lines with a strong necrosis response towards ablative γ-irradiation release monocyte migration factors. Pearson correlation analysis of transmigration (Figures 3A and 6E) and necrosis (Figures 2A,B and 6C) 4 days after irradiation (0 Gy, 2 Gy, daily 2 Gy, or 20 Gy) was performed for p53 mutant cells. The percentage of total or primary necrotic cells was used as in (D). **(G)** Conclusions. Ablative γ-irradiation induces a strong necrotic response in fast proliferating breast cancer cell lines. The concomitant release of nucleotides stimulates monocyte migration and chemokinesis. In p53 wildtype, hormone receptor positive MCF7 cells this is impaired due to irradiation-induced upregulation of CD39, which degrades extracellular nucleotides.

In order to examine, whether our findings that fast proliferating, hormone receptor negative HCC1937 breast cancer cells with defective p53 and a robust necrotic response towards ablative γ-irradiation release monocyte migration stimulating nucleotides, are of broader relevance, we analyzed three more cell lines: HCC1806, MDA-MB468, and BT549 cells. These hormone receptor negative breast cancer cell lines with mutant p53 (Figure 6A) exhibited doubling times of 30 h (HCC1806), 51 h (MDA-MB468) or 77 h (BT549), respectively (Figure 6B). Irradiated HCC1806 and MDA-MB468 cells underwent primary necrosis to a strong and comparable degree as HCC1937 cells, whereas in slowly proliferating BT549 cells no significant induction of necrosis was detected (Figure 6C). When combining the results of all cell lines analyzed in the present study, we observed a clear and significant negative correlation between doubling times and necrosis induction by ablative irradiation with 20 Gy. This held true for total as well as primary necrosis in the presence of zVAD-fmk (Figure 6D). Transwell migration assays with THP-1 cells revealed that only supernatants of irradiated HCC1806 and MDA-MB468 but not BT549 cells released monocyte migration stimulating factors. Again, the strongest monocyte migration was observed with supernatants of ablatively irradiated cells (Figure 6E). As expected, the three p53 defective, ER negative cell lines did neither show any basal CD39 surface expression in FACS analyses, nor its irradiation-induced upregulation (data not shown). Finally, we combined the data on THP-1 cell migration and necrosis induction of all p53 mutant, hormone receptor negative breast cancer lines. The cell lines with strong necrosis induction in response to γ-irradiation were the ones, whose supernatants potently stimulated THP-1 cell migration. Pearson correlation analysis revealed a significant positive correlation between the percentage of THP-1 cell migration and the percentage of total necrosis induced. This correlation was even more stringent when only primary necrosis was considered (Figure 6F).

In summary, our study reveals that fast proliferating, hormone receptor negative breast cancer cell lines with defective p53 intensively undergo necrosis in response to γ-irradiation, particularly when applied in an ablative regime at a single dose of 20 Gy. During necrosis, the cells release nucleotides, which efficiently stimulate monocyte migration in a chemokinetic manner. In hormone receptor positive, p53 wildtype cells, like MCF7, this appears to be hampered by irradiation-induced upregulation of CD39, which degrades extracellular nucleotides. Our study opens several questions, including the detailed molecular mechanisms, which orchestrate irradiation-induced upregulation of CD39 and the specific role of p53 and the hormone receptors in this scenario. Moreover, it will be interesting to further characterize the subpopulation of cells, in which CD39 expression is increased in response to irradiation. The biphasic kinetics and the very strong increase 3 days after irradiation, which parallel senescence induction, support the hypothesis that it might be the non-necrotic, surviving senescent cells. Most importantly, the *in vivo* relevance of our findings has to be explored. Future studies have to address the question, whether ablative irradiation can stimulate monocyte migration and intra-tumoral monocyte recruitment *in vivo*. In this regard it will be of special importance to elucidate if the chemokinetic monocyte response that we have observed *in vitro* might be translated into a directional recruitment into the tumor. Intriguingly, different models of sterile injury have revealed that endothelial cells and pericytes can convert the danger signals released by necrotically dying cells into cascades of chemokine gradients and adhesion molecules, which govern the recruitment of monocytes and neutrophils to the site of injury [56,57]. Along the same line, it has been shown for systemic anthracycline therapy that nucleotides released from dying cancer cells stimulate the intra-tumoral recruitment of a CD11c^+^CD11b^+^Ly6C^hi^ monocytic cell type, which can differentiate into highly potent APCs, engulf tumor material, present it to T cells, and thereby initiate a productive anti-tumor immune response [20]. Extracellular nucleotides appear to be of crucial importance in this context, since they do not only contribute to monocyte recruitment, but also support monocyte activation and differentiation as well as their intra-tumoral survival [20,58]. Hence, it is tempting to speculate that local ablative radiotherapy might be utilized for fast proliferating, hormone receptor negative, p53 mutant breast cancer in order to induce anti-tumor immune responses.

## Conclusions

Here, we show that fast proliferating, hormone receptor negative breast cancer cell lines with mutant p53 intensively undergo necrosis in response to γ-irradiation, especially when applied in an ablative setting at a single dose of 20 Gy. These necrotically dying cancer cells release nucleotides, which stimulate monocyte migration and chemokinesis. In contrast, hormone receptor positive cells with wildtype p53 upregulate CD39 ectonucleotidase expression in response to irradiation, and thus do not stimulate monocyte migration. Future studies have to clarify, whether ablative radiotherapy might be utilized for local necrosis induction and concomitant nucleotide release by the dying cancer cells in order to achieve intra-tumoral monocyte recruitment, APC differentiation, and subsequent priming of adaptive anti-tumor immune responses - not only in the context of fast proliferating, hormone receptor negative and p53 mutant breast cancer.

## Abbreviations

APC: Antigen presenting cell; apyrase: Nucleotide diphosphohydrolase; C12-FDG-FITC: 5-dodecanoylaminofluorescein-di-β-galactopyranoside; FCS: Fetal calf serum; FMI: Forward migration index; PI: Propidium iodide; PS: Phosphatidylserine; qRT-PCR: Quantitative realtime RT-PCR; SA-β-gal: Senescence-associated β-galactosidase; zVAD-fmk: Carbobenzoxy-valyl-alanyl-aspartyl-[O-methyl]-fluoromethylketone.

## Competing interests

The authors declare no conflict of interest.

## Authors’ contributions

KL, and CB conceived and designed experiments. RH, NB, KS, and AE performed experiments and analyzed the data. KL, CB, RH, and HS wrote the manuscript. All authors discussed the results and commented on the manuscript. All authors discussed the results, read and approved the final manuscript.

## References

[B1] Darby S McGale P Correa C Taylor C Arriagada R Clarke M Cutter D Davies C Ewertz M Godwin J Gray R Pierce L Whelan T Wang Y Peto R Effect of radiotherapy after breast-conserving surgery on 10-year recurrence and 15-year breast cancer death: meta-analysis of individual patient data for 10,801 women in 17 randomised trials Lancet 2011 378 9804 1707 1716 22019144 10.1016/S0140-6736(11)61629-2 PMC3254252

[B2] Van Limbergen E Weltens C New trends in radiotherapy for breast cancer Curr Opin Oncol 2006 18 6 555 562 10.1097/01.cco.0000245327.42281.9f 16988575

[B3] Budach W Radiotherapy in patients with metastatic breast cancer Eur J Cancer 2011 47 Suppl 3 S23 S27 21943979 10.1016/S0959-8049(11)70143-5

[B4] Budach W Kammers K Boelke E Matuschek C Adjuvant radiotherapy of regional lymph nodes in breast cancer - a meta-analysis of randomized trials Radiat Oncol 2013 8 1 267 10.1186/1748-717X-8-267 24225206 PMC3842771

[B5] Orth M Lauber K Niyazi M Friedl AA Li M Maihofer C Schuttrumpf L Ernst A Niemoller OM Belka C Current concepts in clinical radiation oncology Radiat Environ Biophys 2014 53 1 1 29 10.1007/s00411-013-0497-2 24141602 PMC3935099

[B6] Pajonk F Vlashi E McBride WH Radiation resistance of cancer stem cells: the 4 R’s of radiobiology revisited Stem Cells 2010 28 4 639 648 10.1002/stem.318 20135685 PMC2940232

[B7] Bentzen SM Agrawal RK Aird EG Barrett JM Barrett-Lee PJ Bliss JM Brown J Dewar JA Dobbs HJ Haviland JS Hoskin PJ Hopwood P Lawton PA Magee BJ Mills J Morgan DA Owen JR Simmons S Sumo G Sydenham MA Venables K Yarnold JR The UK Standardisation of Breast Radiotherapy (START) Trial A of radiotherapy hypofractionation for treatment of early breast cancer: a randomised trial Lancet Oncol 2008 9 4 331 341 18356109 10.1016/S1470-2045(08)70077-9 PMC2323709

[B8] Bentzen SM Agrawal RK Aird EG Barrett JM Barrett-Lee PJ Bliss JM Brown J Dewar JA Dobbs HJ Haviland JS Hoskin PJ Hopwood P Lawton PA Magee BJ Mills J Morgan DA Owen JR Simmons S Sumo G Sydenham MA Venables K Yarnold JR The UK Standardisation of Breast Radiotherapy (START) Trial B of radiotherapy hypofractionation for treatment of early breast cancer: a randomised trial Lancet 2008 371 9618 1098 1107 18355913 10.1016/S0140-6736(08)60348-7 PMC2277488

[B9] Vaidya JS Tobias JS Baum M Keshtgar M Joseph D Wenz F Houghton J Saunders C Corica T D’Souza D Sainsbury R Massarut S Taylor I Hilaris B Intraoperative radiotherapy for breast cancer Lancet Oncol 2004 5 3 165 173 10.1016/S1470-2045(04)01412-3 15003199

[B10] Tuschy B Berlit S Romero S Sperk E Wenz F Kehl S Sutterlin M Clinical aspects of intraoperative radiotherapy in early breast cancer: short-term complications after IORT in women treated with low energy x-rays Radiat Oncol 2013 8 95 10.1186/1748-717X-8-95 23607703 PMC3643839

[B11] Welzel G Boch A Sperk E Hofmann F Kraus-Tiefenbacher U Gerhardt A Suetterlin M Wenz F Radiation-related quality of life parameters after targeted intraoperative radiotherapy versus whole breast radiotherapy in patients with breast cancer: results from the randomized phase III trial TARGIT-A Radiat Oncol 2013 8 1 9 10.1186/1748-717X-8-9 23294485 PMC3896671

[B12] Frey B Rubner Y Kulzer L Werthmoller N Weiss EM Fietkau R Gaipl US Antitumor immune responses induced by ionizing irradiation and further immune stimulation Cancer Immunol Immunother 2014 63 1 29 36 10.1007/s00262-013-1474-y 24052136 PMC11028436

[B13] Lugade AA Moran JP Gerber SA Rose RC Frelinger JG Lord EM Local radiation therapy of B16 melanoma tumors increases the generation of tumor antigen-specific effector cells that traffic to the tumor J Immunol 2005 174 12 7516 7523 15944250 10.4049/jimmunol.174.12.7516

[B14] Lugade AA Sorensen EW Gerber SA Moran JP Frelinger JG Lord EM Radiation-induced IFN-gamma production within the tumor microenvironment influences antitumor immunity J Immunol 2008 180 5 3132 3139 18292536 10.4049/jimmunol.180.5.3132

[B15] Lee Y Auh SL Wang Y Burnette B Meng Y Beckett M Sharma R Chin R Tu T Weichselbaum RR Fu YX Therapeutic effects of ablative radiation on local tumor require CD8+ T cells: changing strategies for cancer treatment Blood 2009 114 3 589 595 10.1182/blood-2009-02-206870 19349616 PMC2713472

[B16] Burnette BC Liang H Lee Y Chlewicki L Khodarev NN Weichselbaum RR Fu YX Auh SL The efficacy of radiotherapy relies upon induction of type i interferon-dependent innate and adaptive immunity Cancer Res 2011 71 7 2488 2496 10.1158/0008-5472.CAN-10-2820 21300764 PMC3070872

[B17] Gupta A Probst HC Vuong V Landshammer A Muth S Yagita H Schwendener R Pruschy M Knuth A van den Broek M Radiotherapy promotes tumor-specific effector CD8+ T cells via dendritic cell activation J Immunol 2012 189 2 558 566 10.4049/jimmunol.1200563 22685313

[B18] Lauber K Ernst A Orth M Herrmann M Belka C Dying cell clearance and its impact on the outcome of tumor radiotherapy Front Oncol 2012 2 116 22973558 10.3389/fonc.2012.00116 PMC3438527

[B19] Apetoh L Ghiringhelli F Tesniere A Obeid M Ortiz C Criollo A Mignot G Maiuri MC Ullrich E Saulnier P Yang H Amigorena S Ryffel B Barrat FJ Saftig P Levi F Lidereau R Nogues C Mira JP Chompret A Joulin V Clavel-Chapelon F Bourhis J Andre F Delaloge S Tursz T Kroemer G Zitvogel L Toll-like receptor 4-dependent contribution of the immune system to anticancer chemotherapy and radiotherapy Nat Med 2007 13 9 1050 1059 10.1038/nm1622 17704786

[B20] Ma Y Adjemian S Mattarollo SR Yamazaki T Aymeric L Yang H Portela Catani JP Hannani D Duret H Steegh K Martins I Schlemmer F Michaud M Kepp O Sukkurwala AQ Menger L Vacchelli E Droin N Galluzzi L Krzysiek R Gordon S Taylor PR Van Endert P Solary E Smyth MJ Zitvogel L Kroemer G Anticancer chemotherapy-induced intratumoral recruitment and differentiation of antigen-presenting cells Immunity 2013 38 4 729 741 10.1016/j.immuni.2013.03.003 23562161

[B21] Lauber K Keppeler H Munoz LE Koppe U Schroder K Yamaguchi H Kronke G Uderhardt S Wesselborg S Belka C Nagata S Herrmann M Milk fat globule-EGF factor 8 mediates the enhancement of apoptotic cell clearance by glucocorticoids Cell Death Differ 2013 20 9 1230 1240 10.1038/cdd.2013.82 23832117 PMC3741508

[B22] Blume KE Soeroes S Keppeler H Stevanovic S Kretschmer D Rautenberg M Wesselborg S Lauber K Cleavage of annexin A1 by ADAM10 during secondary necrosis generates a monocytic “find-me” signal J Immunol 2012 188 1 135 145 10.4049/jimmunol.1004073 22116825

[B23] Peter C Waibel M Keppeler H Lehmann R Xu G Halama A Adamski J Schulze-Osthoff K Wesselborg S Lauber K Release of lysophospholipid ‘find-me’ signals during apoptosis requires the ATP-binding cassette transporter A1 Autoimmunity 2012 45 8 568 573 10.3109/08916934.2012.719947 22913458

[B24] Rosenwald M Koppe U Keppeler H Sauer G Hennel R Ernst A Blume KE Peter C Herrmann M Belka C Schulze-Osthoff K Wesselborg S Lauber K Serum-derived plasminogen is activated by apoptotic cells and promotes their phagocytic clearance J Immunol 2012 189 12 5722 5728 10.4049/jimmunol.1200922 23150713

[B25] Debacq-Chainiaux F Erusalimsky JD Campisi J Toussaint O Protocols to detect senescence-associated beta-galactosidase (SA-betagal) activity, a biomarker of senescent cells in culture and in vivo Nat Protoc 2009 4 12 1798 1806 10.1038/nprot.2009.191 20010931

[B26] Wagner BJ Lindau D Ripper D Stierhof YD Glatzle J Witte M Beck H Keppeler H Lauber K Rammensee HG Konigsrainer A Phagocytosis of dying tumor cells by human peritoneal mesothelial cells J Cell Sci 2011 124 Pt 10 1644 1654 21525033 10.1242/jcs.078907

[B27] Harre U Keppeler H Ipseiz N Derer A Poller K Aigner M Schett G Herrmann M Lauber K Moonlighting osteoclasts as undertakers of apoptotic cells Autoimmunity 2012 48 8 612 619 22978425 10.3109/08916934.2012.719950

[B28] Lauber K Munoz LE Berens C Jendrossek V Belka C Herrmann M Apoptosis induction and tumor cell repopulation: the yin and yang of radiotherapy Radiat Oncol 2011 6 176 10.1186/1748-717X-6-176 22182804 PMC3264523

[B29] Kao J Salari K Bocanegra M Choi YL Girard L Gandhi J Kwei KA Hernandez-Boussard T Wang P Gazdar AF Minna JD Pollack JR Molecular profiling of breast cancer cell lines defines relevant tumor models and provides a resource for cancer gene discovery PLoS One 2009 4 7 e6146 10.1371/journal.pone.0006146 19582160 PMC2702084

[B30] Lacroix M Toillon RA Leclercq G p53 and breast cancer, an update Endocr Relat Cancer 2006 13 2 293 325 10.1677/erc.1.01172 16728565

[B31] Fei P El-Deiry WS P53 and radiation responses Oncogene 2003 22 37 5774 5783 10.1038/sj.onc.1206677 12947385

[B32] Miyashita T Reed JC Tumor suppressor p53 is a direct transcriptional activator of the human bax gene Cell 1995 80 2 293 299 10.1016/0092-8674(95)90412-3 7834749

[B33] Nakano K Vousden KH PUMA, a novel proapoptotic gene, is induced by p53 Mol Cell 2001 7 3 683 694 10.1016/S1097-2765(01)00214-3 11463392

[B34] Oda E Ohki R Murasawa H Nemoto J Shibue T Yamashita T Tokino T Taniguchi T Tanaka N Noxa, a BH3-only member of the Bcl-2 family and candidate mediator of p53-induced apoptosis Science 2000 288 5468 1053 1058 10.1126/science.288.5468.1053 10807576

[B35] Mihara M Erster S Zaika A Petrenko O Chittenden T Pancoska P Moll UM p53 has a direct apoptogenic role at the mitochondria Mol Cell 2003 11 3 577 590 10.1016/S1097-2765(03)00050-9 12667443

[B36] Rufini A Tucci P Celardo I Melino G Senescence and aging: the critical roles of p53 Oncogene 2013 32 43 5129 5143 10.1038/onc.2012.640 23416979

[B37] Jones KR Elmore LW Jackson-Cook C Demasters G Povirk LF Holt SE Gewirtz DA p53-Dependent accelerated senescence induced by ionizing radiation in breast tumour cells Int J Radiat Biol 2005 81 6 445 458 10.1080/09553000500168549 16308915

[B38] Ha L Ichikawa T Anver M Dickins R Lowe S Sharpless NE Krimpenfort P Depinho RA Bennett DC Sviderskaya EV Merlino G ARF functions as a melanoma tumor suppressor by inducing p53-independent senescence Proc Natl Acad Sci U S A 2007 104 26 10968 10973 10.1073/pnas.0611638104 17576930 PMC1904138

[B39] Burnette B Fu YX Weichselbaum RR The confluence of radiotherapy and immunotherapy Front Oncol 2012 2 143 23087904 10.3389/fonc.2012.00143 PMC3472545

[B40] Berens C Lauber K Herrmann M Autoimmunity vs. cancer: predator vs. alien? Autoimmunity 2013 46 5 287 293 10.3109/08916934.2013.787687 23706137

[B41] Peter C Wesselborg S Herrmann M Lauber K Dangerous attraction: phagocyte recruitment and danger signals of apoptotic and necrotic cells Apoptosis 2010 15 9 1007 1028 10.1007/s10495-010-0472-1 20157780

[B42] Ghiringhelli F Apetoh L Tesniere A Aymeric L Ma Y Ortiz C Vermaelen K Panaretakis T Mignot G Ullrich E Perfettini JL Schlemmer F Tasdemir E Uhl M Genin P Civas A Ryffel B Kanellopoulos J Tschopp J Andre F Lidereau R McLaughlin NM Haynes NM Smyth MJ Kroemer G Zitvogel L Activation of the NLRP3 inflammasome in dendritic cells induces IL-1beta-dependent adaptive immunity against tumors Nat Med 2009 15 10 1170 1178 10.1038/nm.2028 19767732

[B43] Elliott MR Chekeni FB Trampont PC Lazarowski ER Kadl A Walk SF Park D Woodson RI Ostankovich M Sharma P Lysiak JJ Harden TK Leitinger N Ravichandran KS Nucleotides released by apoptotic cells act as a find-me signal to promote phagocytic clearance Nature 2009 461 7261 282 286 10.1038/nature08296 19741708 PMC2851546

[B44] Isfort K Ebert F Bornhorst J Sargin S Kardakaris R Pasparakis M Bahler M Schwerdtle T Schwab A Hanley PJ Real-time imaging reveals that P2Y2 and P2Y12 receptor agonists are not chemoattractants and macrophage chemotaxis to complement C5a is phosphatidylinositol 3-kinase (PI3K)- and p38 mitogen-activated protein kinase (MAPK)-independent J Biol Chem 2011 286 52 44776 44787 10.1074/jbc.M111.289793 22057273 PMC3247992

[B45] Kronlage M Song J Sorokin L Isfort K Schwerdtle T Leipziger J Robaye B Conley PB Kim HC Sargin S Schon P Schwab A Hanley PJ Autocrine purinergic receptor signaling is essential for macrophage chemotaxis Sci Signal 2010 3 132 ra55 20664064 10.1126/scisignal.2000588

[B46] Chekeni FB Elliott MR Sandilos JK Walk SF Kinchen JM Lazarowski ER Armstrong AJ Penuela S Laird DW Salvesen GS Isakson BE Bayliss DA Ravichandran KS Pannexin 1 channels mediate ‘find-me’ signal release and membrane permeability during apoptosis Nature 2010 467 7317 863 867 10.1038/nature09413 20944749 PMC3006164

[B47] Antonioli L Pacher P Vizi ES Hasko G CD39 and CD73 in immunity and inflammation Trends Mol Med 2013 19 6 355 367 10.1016/j.molmed.2013.03.005 23601906 PMC3674206

[B48] Clayton A Al-Taei S Webber J Mason MD Tabi Z Cancer exosomes express CD39 and CD73, which suppress T cells through adenosine production J Immunol 2011 187 2 676 683 10.4049/jimmunol.1003884 21677139

[B49] Berger CE Qian Y Liu G Chen H Chen X p53, a target of estrogen receptor (ER) alpha, modulates DNA damage-induced growth suppression in ER-positive breast cancer cells J Biol Chem 2012 287 36 30117 30127 10.1074/jbc.M112.367326 22787161 PMC3436267

[B50] Angeloni SV Martin MB Garcia-Morales P Castro-Galache MD Ferragut JA Saceda M Regulation of estrogen receptor-alpha expression by the tumor suppressor gene p53 in MCF-7 cells J Endocrinol 2004 180 3 497 504 10.1677/joe.0.1800497 15012604

[B51] Menendez D Inga A Resnick MA Estrogen receptor acting in cis enhances WT and mutant p53 transactivation at canonical and noncanonical p53 target sequences Proc Natl Acad Sci U S A 2010 107 4 1500 1505 10.1073/pnas.0909129107 20080630 PMC2824383

[B52] Lion M Bisio A Tebaldi T De Sanctis V Menendez D Resnick MA Ciribilli Y Inga A Interaction between p53 and estradiol pathways in transcriptional responses to chemotherapeutics Cell Cycle 2013 12 8 1211 1224 10.4161/cc.24309 23518503 PMC3674086

[B53] Hallahan DE Dunphy E Virudachalam S Sukhatme VP Kufe DW Weichselbaum RR C-jun and Egr-1 participate in DNA synthesis and cell survival in response to ionizing radiation exposure J Biol Chem 1995 270 51 30303 30309 10.1074/jbc.270.51.30303 8530452

[B54] Lu S Becker KA Hagen MJ Yan H Roberts AL Mathews LA Schneider SS Siegelmann HT MacBeth KJ Tirrell SM Blanchard JL Jerry DJ Transcriptional responses to estrogen and progesterone in mammary gland identify networks regulating p53 activity Endocrinology 2008 149 10 4809 4820 10.1210/en.2008-0035 18556351 PMC2582927

[B55] Liu J Grogan L Nau MM Allegra CJ Chu E Wright JJ Physical interaction between p53 and primary response gene Egr-1 Int J Oncol 2001 18 4 863 870 11251186 10.3892/ijo.18.4.863

[B56] Stark K Eckart A Haidari S Tirniceriu A Lorenz M von Bruhl ML Gartner F Khandoga AG Legate KR Pless R Hepper I Lauber K Walzog B Massberg S Capillary and arteriolar pericytes attract innate leukocytes exiting through venules and ‘instruct’ them with pattern-recognition and motility programs Nat Immunol 2013 14 1 41 51 23179077 10.1038/ni.2477

[B57] McDonald B Pittman K Menezes GB Hirota SA Slaba I Waterhouse CC Beck PL Muruve DA Kubes P Intravascular danger signals guide neutrophils to sites of sterile inflammation Science 2010 330 6002 362 366 10.1126/science.1195491 20947763

[B58] Ma Y Adjemian S Yang H Catani JP Hannani D Martins I Michaud M Kepp O Sukkurwala AQ Vacchelli E Galluzzi L Zitvogel L Kroemer G ATP-dependent recruitment, survival and differentiation of dendritic cell precursors in the tumor bed after anticancer chemotherapy Oncoimmunology 2013 2 6 e24568 10.4161/onci.24568 23894718 PMC3716753

